# Supplementary material for: Comparative multiomics analysis of cell physiological state after culture in a basket bioreactor
Source: Sci Rep. 2022 Nov 23;12:20161. doi: 10.1038/s41598-022-24687-4 (PMC9686226; doi:10.1038/s41598-022-24687-4)
Supplement: Supplementary file 1 — Supplementary Information 1. [file 41598_2022_24687_MOESM1_ESM.zip › raw data/Proteomic raw data/6.KEGGenrichment/EG.vs.CG/ALL/pathway/KEGG.map.html]

Pathway Enrichment


  

# The most enriched pathway terms of EG.vs.CG

Statistic method: hypergeometric test

FDR correction method: Benjamini and Hochberg

| MapID | MapTitle | Pvalue | AdjustedPv | x | y | n | N | EnrichDirect | ProtID | Description  | |
| --- | --- | --- | --- | --- | --- | --- | --- | --- | --- | --- | --- |
| map04514 | Cell adhesion molecules (CAMs) | 3.4089918852652e-06 | 0.000620436523118266 | 10 | 44 | 108 | 2848 | Over | A0A0D9R3G8 A0A0D9R457 A0A0D9R6A4 A0A0D9R9F6 A0A0D9RNI2 A0A0D9RQI3 A0A0D9RYA9 A0A0D9S2Z2 A0A0D9S368 A0A0D9S6M6 | Uncharacterized protein,Uncharacterized protein,Uncharacterized protein,Uncharacterized protein,Ig-like domain-containing protein,B-cell surface antigen CD40,Protein-tyrosine-phosphatase,Neural cell adhesion molecule 1,Uncharacterized protein,Uncharacterized protein |
| map00600 | Sphingolipid metabolism | 0.000399645985287154 | 0.0250357065081722 | 6 | 27 | 108 | 2848 | Over | A0A0D9QUW7 A0A0D9QVQ6 A0A0D9QWC2 A0A0D9RHA9 A0A0D9RVZ2 A0A0D9S760 | Glucuronosyltransferase,Sphingomyelin phosphodiesterase,Sphingomyelin phosphodiesterase,Aminotran\_1\_2 domain-containing protein,Ceramidase,Phospholipid phosphatase 3 |
| map04610 | Complement and coagulation cascades | 0.000412676480903938 | 0.0250357065081722 | 5 | 18 | 108 | 2848 | Over | A0A0D9QZZ6 A0A0D9REV5 A0A0D9RFF4 A0A0D9RRB2 A0A0D9RTW6 | VWFA domain-containing protein,SERPIN domain-containing protein,Coagulation factor V,Plasminogen activator,Clusterin |
| map04512 | ECM-receptor interaction | 0.00935388969749707 | 0.405981493721084 | 5 | 35 | 108 | 2848 | Over | A0A0D9R486 A0A0D9R9X4 A0A0D9RY11 A0A0D9RY12 A0A0D9S8B1 | Collagen type IV alpha 3 chain,Fibronectin,Collagen type IV alpha 2 chain,Collagen IV NC1 domain-containing protein,Uncharacterized protein |
| map04974 | Protein digestion and absorption | 0.0115633189829335 | 0.405981493721084 | 4 | 24 | 108 | 2848 | Over | A0A0D9R486 A0A0D9RY11 A0A0D9RY12 A0A0D9S6M1 | Collagen type IV alpha 3 chain,Collagen type IV alpha 2 chain,Collagen IV NC1 domain-containing protein,Fibrillar collagen NC1 domain-containing protein |
| map03030 | DNA replication | 0.0153509363773452 | 0.405981493721084 | 4 | 26 | 108 | 2848 | Over | A0A0D9RKU0 A0A0D9RLR8 A0A0D9RQN8 A0A0D9RWI0 | DNA replication licensing factor MCM3,Proliferating cell nuclear antigen,DNA replication licensing factor MCM4,Replication factor C subunit 1 |
| map00910 | Nitrogen metabolism | 0.0193413650074991 | 0.405981493721084 | 2 | 6 | 108 | 2848 | Over | A0A0D9RIT7 A0A0D9RQX6 | Carbonic anhydrase,Carbonic anhydrase |
| map04672 | Intestinal immune network for IgA production | 0.0193413650074991 | 0.405981493721084 | 2 | 6 | 108 | 2848 | Over | A0A0D9R6A4 A0A0D9RQI3 | Uncharacterized protein,B-cell surface antigen CD40 |
| map04110 | Cell cycle | 0.0210787046451195 | 0.405981493721084 | 6 | 58 | 108 | 2848 | Over | A0A0D9REV4 A0A0D9RJF7 A0A0D9RKU0 A0A0D9RLR8 A0A0D9RQN8 A0A0D9RYS3 | Uncharacterized protein,Structural maintenance of chromosomes protein 1A,DNA replication licensing factor MCM3,Proliferating cell nuclear antigen,DNA replication licensing factor MCM4,Uncharacterized protein |
| map05146 | Amoebiasis | 0.0239080611487494 | 0.405981493721084 | 5 | 44 | 108 | 2848 | Over | A0A0D9R486 A0A0D9R9X4 A0A0D9RQT0 A0A0D9RY11 A0A0D9RY12 | Collagen type IV alpha 3 chain,Fibronectin,Uncharacterized protein,Collagen type IV alpha 2 chain,Collagen IV NC1 domain-containing protein |
| map05222 | Small cell lung cancer | 0.0261075977272294 | 0.405981493721084 | 5 | 45 | 108 | 2848 | Over | A0A0D9R486 A0A0D9R9X4 A0A0D9RY11 A0A0D9RY12 A0A0D9RYS3 | Collagen type IV alpha 3 chain,Fibronectin,Collagen type IV alpha 2 chain,Collagen IV NC1 domain-containing protein,Uncharacterized protein |
| map04151 | PI3K-Akt signaling pathway | 0.0270332176427469 | 0.405981493721084 | 10 | 127 | 108 | 2848 | Over | A0A0D9QZT6 A0A0D9R2B7 A0A0D9R486 A0A0D9R491 A0A0D9R6H1 A0A0D9R9X4 A0A0D9RY11 A0A0D9RY12 A0A0D9S336 A0A0D9S6I0 | RRM domain-containing protein,Uncharacterized protein,Collagen type IV alpha 3 chain,Uncharacterized protein,Glycogen [starch] synthase,Fibronectin,Collagen type IV alpha 2 chain,Collagen IV NC1 domain-containing protein,Glycogen [starch] synthase,Macrophage colony-stimulating factor 1 |
| map00500 | Starch and sucrose metabolism | 0.0330158905445515 | 0.405981493721084 | 3 | 19 | 108 | 2848 | Over | A0A0D9R6H1 A0A0D9RT09 A0A0D9S336 | Glycogen [starch] synthase,Uncharacterized protein,Glycogen [starch] synthase |
| map04933 | AGE-RAGE signaling pathway in diabetic complications | 0.0334691412649148 | 0.405981493721084 | 5 | 48 | 108 | 2848 | Over | A0A0D9R486 A0A0D9R9X4 A0A0D9RY11 A0A0D9RY12 A0A0D9S6M6 | Collagen type IV alpha 3 chain,Fibronectin,Collagen type IV alpha 2 chain,Collagen IV NC1 domain-containing protein,Uncharacterized protein |
| map00072 | Synthesis and degradation of ketone bodies | 0.0343564801943533 | 0.405981493721084 | 2 | 8 | 108 | 2848 | Over | A0A0D9RJH6 A0A0D9S9P1 | Uncharacterized protein,Uncharacterized protein |
| map04979 | Cholesterol metabolism | 0.0377967877414668 | 0.405981493721084 | 3 | 20 | 108 | 2848 | Over | A0A0D9RJC1 A0A0D9RRV8 A0A0D9S6J4 | Epididymal secretory protein E1,Bile salt export pump,Sortilin 1 |
| map05310 | Asthma | 0.0379213483146068 | 0.405981493721084 | 1 | 1 | 108 | 2848 | Over | A0A0D9RQI3 | B-cell surface antigen CD40 |
| map04060 | Cytokine-cytokine receptor interaction | 0.0429116640054933 | 0.43388460272221 | 3 | 21 | 108 | 2848 | Over | A0A0D9RQI3 A0A0D9S6I0 A0A0D9S8Z3 | B-cell surface antigen CD40,Macrophage colony-stimulating factor 1,TNF receptor superfamily member 18 |
| map04910 | Insulin signaling pathway | 0.0730795989974692 | 0.677199091888565 | 6 | 78 | 108 | 2848 | Over | A0A0D9R1V2 A0A0D9R2B7 A0A0D9R491 A0A0D9R6H1 A0A0D9RU34 A0A0D9S336 | E3 ubiquitin-protein ligase CBL,Uncharacterized protein,Uncharacterized protein,Glycogen [starch] synthase,CBM21 domain-containing protein,Glycogen [starch] synthase |
| map04950 | Maturity onset diabetes of the young | 0.074417482625117 | 0.677199091888565 | 1 | 2 | 108 | 2848 | Over | A0A0D9QYX8 | Uncharacterized protein |
| map01521 | EGFR tyrosine kinase inhibitor resistance | 0.0887820100830767 | 0.769444087386665 | 4 | 45 | 108 | 2848 | Over | A0A0D9R2B7 A0A0D9R491 A0A0D9RXU0 A0A0D9S0W1 | Uncharacterized protein,Uncharacterized protein,Growth arrest specific 6,Receptor protein-tyrosine kinase |
| map00830 | Retinol metabolism | 0.108565920980014 | 0.859086852972282 | 2 | 15 | 108 | 2848 | Over | A0A0D9RMB0 A0A0D9S8K6 | NADP-retinol dehydrogenase,Uncharacterized protein |
| map05144 | Malaria | 0.108565920980014 | 0.859086852972282 | 2 | 15 | 108 | 2848 | Over | A0A0D9RQI3 A0A0D9S6M6 | B-cell surface antigen CD40,Uncharacterized protein |
| map04064 | NF-kappa B signaling pathway | 0.127506207470182 | 0.86002457223354 | 3 | 33 | 108 | 2848 | Over | A0A0D9RQI3 A0A0D9RWK4 A0A0D9S6M6 | B-cell surface antigen CD40,Ubiquitinyl hydrolase 1,Uncharacterized protein |
| map04668 | TNF signaling pathway | 0.127506207470182 | 0.86002457223354 | 3 | 33 | 108 | 2848 | Over | A0A0D9RWK4 A0A0D9S6I0 A0A0D9S6M6 | Ubiquitinyl hydrolase 1,Macrophage colony-stimulating factor 1,Uncharacterized protein |
| map00900 | Terpenoid backbone biosynthesis | 0.134002001742661 | 0.871013011327294 | 2 | 17 | 108 | 2848 | Over | A0A0D9RJH6 A0A0D9S9P1 | Uncharacterized protein,Uncharacterized protein |
| map05340 | Primary immunodeficiency | 0.143344479971799 | 0.892895531703589 | 1 | 4 | 108 | 2848 | Over | A0A0D9RQI3 | B-cell surface antigen CD40 |
| map00650 | Butanoate metabolism | 0.147180582148943 | 0.892895531703589 | 2 | 18 | 108 | 2848 | Over | A0A0D9RJH6 A0A0D9S9P1 | Uncharacterized protein,Uncharacterized protein |
| map00310 | Lysine degradation | 0.154132144528431 | 0.893881951118991 | 3 | 36 | 108 | 2848 | Over | A0A0D9R3X6 A0A0D9RJH6 A0A0D9S9P1 | Histone-lysine N-methyltransferase EZH2,Uncharacterized protein,Uncharacterized protein |
| map03430 | Mismatch repair | 0.160611722337695 | 0.893881951118991 | 2 | 19 | 108 | 2848 | Over | A0A0D9RLR8 A0A0D9RWI0 | Proliferating cell nuclear antigen,Replication factor C subunit 1 |
| map00380 | Tryptophan metabolism | 0.174257826181721 | 0.893881951118991 | 2 | 20 | 108 | 2848 | Over | A0A0D9RJH6 A0A0D9S9P1 | Uncharacterized protein,Uncharacterized protein |
| map00565 | Ether lipid metabolism | 0.174257826181721 | 0.893881951118991 | 2 | 20 | 108 | 2848 | Over | A0A0D9QUW7 A0A0D9S760 | Glucuronosyltransferase,Phospholipid phosphatase 3 |
| map04978 | Mineral absorption | 0.174257826181721 | 0.893881951118991 | 2 | 20 | 108 | 2848 | Over | A0A0D9RJ61 A0A0D9S334 | Ferric oxidoreductase domain-containing protein,Ferritin |
| map04360 | Axon guidance | 0.188618023440956 | 0.893881951118991 | 5 | 70 | 108 | 2848 | Over | A0A0D9R3G8 A0A0D9R4M5 A0A0D9R710 A0A0D9RQF9 A0A0D9RQM0 | Uncharacterized protein,Uncharacterized protein,Plexin A1,Protein-serine/threonine kinase,Uncharacterized protein |
| map05202 | Transcriptional misregulation in cancer | 0.192027490600182 | 0.893881951118991 | 3 | 40 | 108 | 2848 | Over | A0A0D9RJ75 A0A0D9RQI3 A0A0D9RRB2 | Runt-related transcription factor,B-cell surface antigen CD40,Plasminogen activator |
| map05215 | Prostate cancer | 0.192027490600182 | 0.893881951118991 | 3 | 40 | 108 | 2848 | Over | A0A0D9R491 A0A0D9RRB2 A0A0D9RYS3 | Uncharacterized protein,Plasminogen activator,Uncharacterized protein |
| map01522 | Endocrine resistance | 0.201851553646701 | 0.896023969846329 | 3 | 41 | 108 | 2848 | Over | A0A0D9R491 A0A0D9RRV8 A0A0D9RYS3 | Uncharacterized protein,Bile salt export pump,Uncharacterized protein |
| map05220 | Chronic myeloid leukemia | 0.231984752050003 | 0.955850896496734 | 3 | 44 | 108 | 2848 | Over | A0A0D9R1V2 A0A0D9R491 A0A0D9RYS3 | E3 ubiquitin-protein ligase CBL,Uncharacterized protein,Uncharacterized protein |
| map00730 | Thiamine metabolism | 0.237310747909969 | 0.955850896496734 | 1 | 7 | 108 | 2848 | Over | A0A0D9S8B5 | Alkaline phosphatase |
| map04080 | Neuroactive ligand-receptor interaction | 0.237310747909969 | 0.955850896496734 | 1 | 7 | 108 | 2848 | Over | A0A0D9RJD8 | KISS1 receptor |
| map00630 | Glyoxylate and dicarboxylate metabolism | 0.244560754679411 | 0.955850896496734 | 2 | 25 | 108 | 2848 | Over | A0A0D9RJH6 A0A0D9S9P1 | Uncharacterized protein,Uncharacterized protein |
| map04657 | IL-17 signaling pathway | 0.258835144436602 | 0.955850896496734 | 2 | 26 | 108 | 2848 | Over | A0A0D9RNH8 A0A0D9RWK4 | Lipocalin 2,Ubiquitinyl hydrolase 1 |
| map04976 | Bile secretion | 0.287402644758437 | 0.955850896496734 | 2 | 28 | 108 | 2848 | Over | A0A0D9RQJ5 A0A0D9RRV8 | Aquaporin-1,Bile salt export pump |
| map00920 | Sulfur metabolism | 0.294205322053788 | 0.955850896496734 | 1 | 9 | 108 | 2848 | Over | A0A0D9S1A5 | Lactamase\_B domain-containing protein |
| map05152 | Tuberculosis | 0.304930702842773 | 0.955850896496734 | 4 | 64 | 108 | 2848 | Over | A0A0D9QV27 A0A0D9QZZ6 A0A0D9R491 A0A0D9RBZ8 | Uncharacterized protein,VWFA domain-containing protein,Uncharacterized protein,Cathepsin D |
| map05161 | Hepatitis B | 0.315187864479247 | 0.955850896496734 | 4 | 66 | 108 | 2848 | Over | A0A0D9R491 A0A0D9RLR8 A0A0D9RYS3 A0A0D9S8B1 | Uncharacterized protein,Proliferating cell nuclear antigen,Uncharacterized protein,Uncharacterized protein |
| map05218 | Melanoma | 0.315860601756524 | 0.955850896496734 | 2 | 30 | 108 | 2848 | Over | A0A0D9R491 A0A0D9RYS3 | Uncharacterized protein,Uncharacterized protein |
| map00220 | Arginine biosynthesis | 0.321054855417011 | 0.955850896496734 | 1 | 10 | 108 | 2848 | Over | A0A0D9QZS1 | Alanine transaminase |
| map04975 | Fat digestion and absorption | 0.321054855417011 | 0.955850896496734 | 1 | 10 | 108 | 2848 | Over | A0A0D9S760 | Phospholipid phosphatase 3 |
| map05320 | Autoimmune thyroid disease | 0.321054855417011 | 0.955850896496734 | 1 | 10 | 108 | 2848 | Over | A0A0D9RQI3 | B-cell surface antigen CD40 |
| map05330 | Allograft rejection | 0.321054855417011 | 0.955850896496734 | 1 | 10 | 108 | 2848 | Over | A0A0D9RQI3 | B-cell surface antigen CD40 |
| map04071 | Sphingolipid signaling pathway | 0.326195227250201 | 0.955850896496734 | 4 | 68 | 108 | 2848 | Over | A0A0D9QWC2 A0A0D9RBZ8 A0A0D9RHA9 A0A0D9RVZ2 | Sphingomyelin phosphodiesterase,Cathepsin D,Aminotran\_1\_2 domain-containing protein,Ceramidase |
| map00071 | Fatty acid degradation | 0.330003631351722 | 0.955850896496734 | 2 | 31 | 108 | 2848 | Over | A0A0D9RJH6 A0A0D9S9P1 | Uncharacterized protein,Uncharacterized protein |
| map00640 | Propanoate metabolism | 0.330003631351722 | 0.955850896496734 | 2 | 31 | 108 | 2848 | Over | A0A0D9RJH6 A0A0D9S9P1 | Uncharacterized protein,Uncharacterized protein |
| map04621 | NOD-like receptor signaling pathway | 0.343940986423883 | 0.955850896496734 | 4 | 71 | 108 | 2848 | Over | A0A0D9QWT4 A0A0D9RR35 A0A0D9RWK4 A0A0D9S6T5 | Anthrax toxin receptor,VWFA domain-containing protein,Ubiquitinyl hydrolase 1,GB1/RHD3-type G domain-containing protein |
| map00480 | Glutathione metabolism | 0.344068172236943 | 0.955850896496734 | 2 | 32 | 108 | 2848 | Over | A0A0D9R7G0 A0A0D9RRE1 | Glutathione transferase,Glutathione peroxidase |
| map03420 | Nucleotide excision repair | 0.344068172236943 | 0.955850896496734 | 2 | 32 | 108 | 2848 | Over | A0A0D9RLR8 A0A0D9RWI0 | Proliferating cell nuclear antigen,Replication factor C subunit 1 |
| map05217 | Basal cell carcinoma | 0.371754852777534 | 0.955850896496734 | 1 | 12 | 108 | 2848 | Over | A0A0D9SCP4 | Uncharacterized protein |
| map00620 | Pyruvate metabolism | 0.371905715746973 | 0.955850896496734 | 2 | 34 | 108 | 2848 | Over | A0A0D9RJH6 A0A0D9S9P1 | Uncharacterized protein,Uncharacterized protein |
| map04142 | Lysosome | 0.379256319678468 | 0.955850896496734 | 5 | 86 | 108 | 2848 | Over | A0A0D9QWC2 A0A0D9RBZ8 A0A0D9RJC1 A0A0D9RVZ2 A0A0D9S6J4 | Sphingomyelin phosphodiesterase,Cathepsin D,Epididymal secretory protein E1,Ceramidase,Sortilin 1 |
| map00531 | Glycosaminoglycan degradation | 0.395679562192212 | 0.955850896496734 | 1 | 13 | 108 | 2848 | Over | A0A0D9RP19 | Hyaluronidase |
| map05020 | Prion diseases | 0.395679562192212 | 0.955850896496734 | 1 | 13 | 108 | 2848 | Over | A0A0D9S2Z2 | Neural cell adhesion molecule 1 |
| map05143 | African trypanosomiasis | 0.395679562192212 | 0.955850896496734 | 1 | 13 | 108 | 2848 | Over | A0A0D9S6M6 | Uncharacterized protein |
| map01212 | Fatty acid metabolism | 0.399272466551142 | 0.955850896496734 | 2 | 36 | 108 | 2848 | Over | A0A0D9RJH6 A0A0D9S9P1 | Uncharacterized protein,Uncharacterized protein |
| map05223 | Non-small cell lung cancer | 0.399272466551142 | 0.955850896496734 | 2 | 36 | 108 | 2848 | Over | A0A0D9R491 A0A0D9RYS3 | Uncharacterized protein,Uncharacterized protein |
| map00100 | Steroid biosynthesis | 0.418701293156318 | 0.955850896496734 | 1 | 14 | 108 | 2848 | Over | A0A0D9QXG6 | Cytochrome P450 family 2 subfamily R member 1 |
| map01210 | 2-Oxocarboxylic acid metabolism | 0.418701293156318 | 0.955850896496734 | 1 | 14 | 108 | 2848 | Over | A0A0D9QZS1 | Alanine transaminase |
| map04745 | Phototransduction - fly | 0.418701293156318 | 0.955850896496734 | 1 | 14 | 108 | 2848 | Over | A0A0D9R294 | Lipase\_3 domain-containing protein |
| map04510 | Focal adhesion | 0.419558832593394 | 0.955850896496734 | 5 | 98 | 108 | 2848 | Over | A0A0D9R486 A0A0D9R491 A0A0D9R9X4 A0A0D9RY11 A0A0D9RY12 | Collagen type IV alpha 3 chain,Uncharacterized protein,Fibronectin,Collagen type IV alpha 2 chain,Collagen IV NC1 domain-containing protein |
| map05205 | Proteoglycans in cancer | 0.432818275125962 | 0.955850896496734 | 5 | 101 | 108 | 2848 | Over | A0A0D9QZT6 A0A0D9R1V2 A0A0D9R9X4 A0A0D9S8B1 A0A0D9SCP4 | RRM domain-containing protein,E3 ubiquitin-protein ligase CBL,Fibronectin,Uncharacterized protein,Uncharacterized protein |
| map00760 | Nicotinate and nicotinamide metabolism | 0.44085381974034 | 0.955850896496734 | 1 | 15 | 108 | 2848 | Over | A0A0D9RXR7 | Ecto-5-nucleotidase |
| map00982 | Drug metabolism - cytochrome P450 | 0.44085381974034 | 0.955850896496734 | 1 | 15 | 108 | 2848 | Over | A0A0D9R7G0 | Glutathione transferase |
| map03410 | Base excision repair | 0.44085381974034 | 0.955850896496734 | 1 | 15 | 108 | 2848 | Over | A0A0D9RLR8 | Proliferating cell nuclear antigen |
| map04964 | Proximal tubule bicarbonate reclamation | 0.44085381974034 | 0.955850896496734 | 1 | 15 | 108 | 2848 | Over | A0A0D9RQJ5 | Aquaporin-1 |
| map04926 | Relaxin signaling pathway | 0.44158003752398 | 0.955850896496734 | 3 | 51 | 108 | 2848 | Over | A0A0D9R486 A0A0D9RY11 A0A0D9RY12 | Collagen type IV alpha 3 chain,Collagen type IV alpha 2 chain,Collagen IV NC1 domain-containing protein |
| map04722 | Neurotrophin signaling pathway | 0.446848947073779 | 0.955850896496734 | 3 | 52 | 108 | 2848 | Over | A0A0D9R491 A0A0D9RJT1 A0A0D9S6J4 | Uncharacterized protein,MAGE domain-containing protein,Sortilin 1 |
| map04931 | Insulin resistance | 0.452329915043093 | 0.955850896496734 | 3 | 53 | 108 | 2848 | Over | A0A0D9R6H1 A0A0D9RU34 A0A0D9S336 | Glycogen [starch] synthase,CBM21 domain-containing protein,Glycogen [starch] synthase |
| map00590 | Arachidonic acid metabolism | 0.462169664240179 | 0.955850896496734 | 1 | 16 | 108 | 2848 | Over | A0A0D9RRE1 | Glutathione peroxidase |
| map00670 | One carbon pool by folate | 0.462169664240179 | 0.955850896496734 | 1 | 16 | 108 | 2848 | Over | A0A0D9RSM3 | Methenyltetrahydrofolate cyclohydrolase |
| map04640 | Hematopoietic cell lineage | 0.462169664240179 | 0.955850896496734 | 1 | 16 | 108 | 2848 | Over | A0A0D9S6I0 | Macrophage colony-stimulating factor 1 |
| map05418 | Fluid shear stress and atherosclerosis | 0.482383780279905 | 0.965360286292547 | 3 | 58 | 108 | 2848 | Over | A0A0D9R7G0 A0A0D9RRB2 A0A0D9S6M6 | Glutathione transferase,Plasminogen activator,Uncharacterized protein |
| map00790 | Folate biosynthesis | 0.482680143146274 | 0.965360286292547 | 1 | 17 | 108 | 2848 | Over | A0A0D9S8B5 | Alkaline phosphatase |
| map05219 | Bladder cancer | 0.482680143146274 | 0.965360286292547 | 1 | 17 | 108 | 2848 | Over | A0A0D9RYS3 | Uncharacterized protein |
| map02010 | ABC transporters | 0.502415411440234 | 0.982209197290236 | 1 | 18 | 108 | 2848 | Over | A0A0D9RRV8 | Bile salt export pump |
| map05322 | Systemic lupus erythematosus | 0.502415411440234 | 0.982209197290236 | 1 | 18 | 108 | 2848 | Over | A0A0D9RQI3 | B-cell surface antigen CD40 |
| map04014 | Ras signaling pathway | 0.530374735000703 | 0.982209197290236 | 4 | 75 | 108 | 2848 | Over | A0A0D9R3Z2 A0A0D9R491 A0A0D9RQM0 A0A0D9S6I0 | Lipase domain-containing protein,Uncharacterized protein,Uncharacterized protein,Macrophage colony-stimulating factor 1 |
| map05225 | Hepatocellular carcinoma | 0.530374735000703 | 0.982209197290236 | 4 | 75 | 108 | 2848 | Over | A0A0D9R491 A0A0D9R7G0 A0A0D9RYS3 A0A0D9SCP4 | Uncharacterized protein,Glutathione transferase,Uncharacterized protein,Uncharacterized protein |
| map00250 | Alanine, aspartate and glutamate metabolism | 0.539675383126503 | 0.982209197290236 | 1 | 20 | 108 | 2848 | Over | A0A0D9QZS1 | Alanine transaminase |
| map05014 | Amyotrophic lateral sclerosis (ALS) | 0.539675383126503 | 0.982209197290236 | 1 | 20 | 108 | 2848 | Over | A0A0D9R491 | Uncharacterized protein |
| map00980 | Metabolism of xenobiotics by cytochrome P450 | 0.557254965383341 | 1 | 1 | 21 | 108 | 2848 | Over | A0A0D9R7G0 | Glutathione transferase |
| map05200 | Pathways in cancer | 0.565692571133156 | 1 | 9 | 186 | 108 | 2848 | Over | A0A0D9R1V2 A0A0D9R486 A0A0D9R491 A0A0D9R7G0 A0A0D9R9X4 A0A0D9RY11 A0A0D9RY12 A0A0D9RYS3 A0A0D9SCP4 | E3 ubiquitin-protein ligase CBL,Collagen type IV alpha 3 chain,Uncharacterized protein,Glutathione transferase,Fibronectin,Collagen type IV alpha 2 chain,Collagen IV NC1 domain-containing protein,Uncharacterized protein,Uncharacterized protein |
| map04924 | Renin secretion | 0.574169172577752 | 1 | 1 | 22 | 108 | 2848 | Over | A0A0D9RQJ5 | Aquaporin-1 |
| map05204 | Chemical carcinogenesis | 0.574169172577752 | 1 | 1 | 22 | 108 | 2848 | Over | A0A0D9R7G0 | Glutathione transferase |
| map05166 | HTLV-I infection | 0.59697809447946 | 1 | 5 | 105 | 108 | 2848 | Over | A0A0D9RLR8 A0A0D9RQI3 A0A0D9RYS3 A0A0D9S6M6 A0A0D9SCP4 | Proliferating cell nuclear antigen,B-cell surface antigen CD40,Uncharacterized protein,Uncharacterized protein,Uncharacterized protein |
| map05165 | Human papillomavirus infection | 0.615218856162284 | 1 | 7 | 142 | 108 | 2848 | Over | A0A0D9R486 A0A0D9R491 A0A0D9R9X4 A0A0D9RY11 A0A0D9RY12 A0A0D9RYS3 A0A0D9SCP4 | Collagen type IV alpha 3 chain,Uncharacterized protein,Fibronectin,Collagen type IV alpha 2 chain,Collagen IV NC1 domain-containing protein,Uncharacterized protein,Uncharacterized protein |
| map00330 | Arginine and proline metabolism | 0.621164514348095 | 1 | 1 | 25 | 108 | 2848 | Over | A0A0D9R8E6 | Procollagen-proline 4-dioxygenase |
| map05416 | Viral myocarditis | 0.621164514348095 | 1 | 1 | 25 | 108 | 2848 | Over | A0A0D9RQI3 | B-cell surface antigen CD40 |
| map05120 | Epithelial cell signaling in Helicobacter pylori infection | 0.64975503999108 | 1 | 2 | 37 | 108 | 2848 | Over | A0A0D9R3N7 A0A0D9R9F6 | GIT ArfGAP 1,Uncharacterized protein |
| map04550 | Signaling pathways regulating pluripotency of stem cells | 0.662984919534136 | 1 | 2 | 40 | 108 | 2848 | Over | A0A0D9QYX8 A0A0D9SCP4 | Uncharacterized protein,Uncharacterized protein |
| map01524 | Platinum drug resistance | 0.672593372696277 | 1 | 2 | 42 | 108 | 2848 | Over | A0A0D9R491 A0A0D9R7G0 | Uncharacterized protein,Glutathione transferase |
| map04012 | ErbB signaling pathway | 0.672593372696277 | 1 | 2 | 42 | 108 | 2848 | Over | A0A0D9R1V2 A0A0D9R491 | E3 ubiquitin-protein ligase CBL,Uncharacterized protein |
| map00280 | Valine, leucine and isoleucine degradation | 0.677580092446558 | 1 | 2 | 43 | 108 | 2848 | Over | A0A0D9RJH6 A0A0D9S9P1 | Uncharacterized protein,Uncharacterized protein |
| map05212 | Pancreatic cancer | 0.6826670640842 | 1 | 2 | 44 | 108 | 2848 | Over | A0A0D9R491 A0A0D9RYS3 | Uncharacterized protein,Uncharacterized protein |
| map05224 | Breast cancer | 0.687839516863725 | 1 | 2 | 45 | 108 | 2848 | Over | A0A0D9RYS3 A0A0D9SCP4 | Uncharacterized protein,Uncharacterized protein |
| map04670 | Leukocyte transendothelial migration | 0.698386555062061 | 1 | 2 | 47 | 108 | 2848 | Over | A0A0D9R9F6 A0A0D9S6M6 | Uncharacterized protein,Uncharacterized protein |
| map05100 | Bacterial invasion of epithelial cells | 0.698386555062061 | 1 | 2 | 47 | 108 | 2848 | Over | A0A0D9R1V2 A0A0D9R9X4 | E3 ubiquitin-protein ligase CBL,Fibronectin |
| map04922 | Glucagon signaling pathway | 0.709121431819286 | 1 | 2 | 49 | 108 | 2848 | Over | A0A0D9R6H1 A0A0D9S336 | Glycogen [starch] synthase,Glycogen [starch] synthase |
| map04310 | Wnt signaling pathway | 0.71453175494802 | 1 | 2 | 50 | 108 | 2848 | Over | A0A0D9R633 A0A0D9SCP4 | Uncharacterized protein,Uncharacterized protein |
| map05145 | Toxoplasmosis | 0.71453175494802 | 1 | 2 | 50 | 108 | 2848 | Over | A0A0D9R491 A0A0D9RQI3 | Uncharacterized protein,B-cell surface antigen CD40 |
| map04217 | Necroptosis | 0.742603069691129 | 1 | 3 | 68 | 108 | 2848 | Over | A0A0D9QWC2 A0A0D9RWK4 A0A0D9S334 | Sphingomyelin phosphodiesterase,Ubiquitinyl hydrolase 1,Ferritin |
| map04140 | Autophagy - animal | 0.755546621631625 | 1 | 3 | 73 | 108 | 2848 | Over | A0A0D9R491 A0A0D9RBZ8 A0A0D9RQT0 | Uncharacterized protein,Cathepsin D,Uncharacterized protein |
| map04115 | p53 signaling pathway | 1 | 1 | 1 | 26 | 108 | 2848 | Over | A0A0D9QZU3 | Tetraspanin |
| map04727 | GABAergic synapse | 1 | 1 | 1 | 26 | 108 | 2848 | Over | A0A0D9R329 | Aa\_trans domain-containing protein |
| map05323 | Rheumatoid arthritis | 1 | 1 | 1 | 26 | 108 | 2848 | Over | A0A0D9S6I0 | Macrophage colony-stimulating factor 1 |
